# Thermal Decomposition Path—Studied by the Simultaneous Thermogravimetry Coupled with Fourier Transform Infrared Spectroscopy and Quadrupole Mass Spectrometry—Of Imidazoline/Dimethyl Succinate Hybrids and Their Biological Characterization

**DOI:** 10.3390/ma16134638

**Published:** 2023-06-27

**Authors:** Marta Worzakowska, Małgorzata Sztanke, Jolanta Rzymowska, Krzysztof Sztanke

**Affiliations:** 1 Department of Polymer Chemistry, Institute of Chemical Sciences, Faculty of Chemistry, Maria Curie-Skłodowska University in Lublin, 33 Gliniana Street, 20-614 Lublin, Poland; 2 Department of Medical Chemistry, Medical University of Lublin, 4A Chodźki Street, 20-093 Lublin, Poland; malgorzata.sztanke@umlub.pl; 3 Department of Biology and Genetics, Medical University of Lublin, 4A Chodźki Street, 20-093 Lublin, Poland; jolanta.rzymowska@umlub.pl; 4 Laboratory of Bioorganic Compounds Synthesis and Analysis, Medical University of Lublin, 4A Chodźki Street, 20-093 Lublin, Poland; krzysztof.sztanke@umlub.pl

**Keywords:** imidazoline/dimethyl succinate hybrids, decomposition course, thermal behavior, antitumor activity, antihemolytic activity, toxicity to normal cells

## Abstract

The thermal decomposition path of synthetically and pharmacologically useful hybrid materials was analyzed in inert and oxidizing conditions for the first time and presented in this article. All the imidazoline/dimethyl succinate hybrids (**1**–**5**) were studied using the simultaneous thermogravimetry (TG) coupled with Fourier transform infrared spectroscopy (FTIR) and quadrupole mass spectrometry (QMS). It was found that the tested compounds were thermally stable up to 200–208 °C (inert conditions) and up to 191–197 °C (oxidizing conditions). In both furnace atmospheres, their decomposition paths were multi-step processes. At least two major stages (inert conditions) and three major stages (oxidizing conditions) of their decomposition were observed. The first decomposition stage occurred between *T*_5%_ and 230–237 °C. It was connected with the breaking of one ester bond. This led to the emission of one methanol molecule and the formation of radicals capable of further radical reactions in both used atmospheres. At the second decomposition stage (*T*_max2_) between 230–237 °C and 370 °C (inert conditions), or at about 360 °C (oxidizing conditions), the cleavage of the second ester bond and N-N and C-C bonds led to the emission of CH_3_OH, HCN, N_2_, and CO_2_ and other radical fragments that reacted with each other to form clusters and large clusters. Heating the tested compounds to a temperature of about 490 °C resulted in the emission of NH_3_, HCN, HNCO, aromatic amines, carbonyl fragments, and the residue (*T*_max2a_) in both atmospheres. In oxidizing conditions, the oxidation of the formed residues (*T*_max3_) was related to the production of CO_2_, CO, and H_2_O. These studies confirmed the same radical decomposition mechanism of the tested compounds both in inert and oxidizing conditions. The antitumor activities and toxicities to normal cells of the imidazoline/dimethyl succinate hybrids were also evaluated. As a result, the two hybrid materials (**3** and **5**) proved to be the most selective in biological studies, and therefore, they should be utilized in further, more extended in vivo investigations.

## 1. Introduction

**Figure 1:**
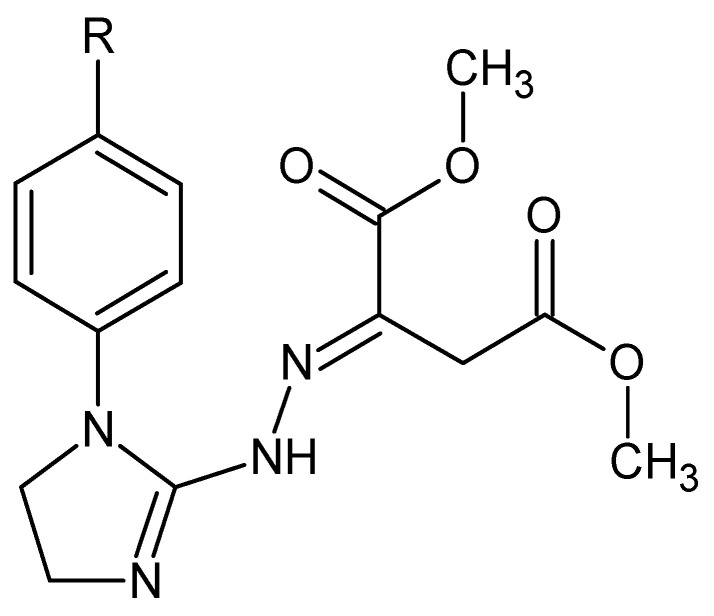
Structures of the investigated hybrid materials, i.e., dimethyl 2-{2-[1-(R-phenyl)-4,5-dihydro-1*H*-imidazol-2-yl]hydrazinylidene}succinates: **1**. R = H, **2**. R = 4-CH_3_, **3**. R = 4-OCH_3_, **4**. R = 4- OCH_2_CH_3_, and **5**. R = 4-Cl.

Dimethyl 2-{2-[1-(R-phenyl)-4,5-dihydro-1*H*-imidazol-2-yl]hydrazinylidene}succinates (**1**–**5**) (Figure 1) constitute a class of imidazoline-based hybrids with dimethyl succinate whose structures in solution are known. The molecules of these less polar, possible diester prodrugs should pass cell membranes more easily than their more polar dicarboxylic acid analogues. All the title compounds found applications as alternative starting materials [1] in the organic synthesis of pharmaceutically important fused triazinones containing the functional moiety of methyl acetate or acetohydrazide at the *C*-3 [2,3].

The literature survey revealed several papers concerning the thermal analysis of succinic acid, sodium succinate, and its esters used in steroidal pharmaceutics. Caires et al. [4,5] reported the thermal stability and combustion processes of succinic acid and its sodium dibasic hexahydrate salt. Mihovec et al. [6] and Trissel and Zhang [7] studied the thermal stability of hydrocortisone sodium succinate and methylprednisolone sodium succinate in their dosage forms, respectively. In turn, the pharmaceutically important imidazoline derivatives were less thermally studied. Ghabbour et al. [8] characterized 2-(2,6-dichlorophenylamino)-2-imidazoline tetraphenylborate—salt of clonidine (an antihypertensive agent) by differential scanning calorimetry and thermogravimetry. Legendre et al. [9] described the polymorphic nature of 2-(2-benzofuryl)-Δ^2^-imidazoline (an antidepressant and vasodilator agent). In turn, Marciniec et al. [10] performed the thermal study of some irradiated imidazoline-containing drugs, such as antazoline and tymazoline hydrochlorides (antihistaminic agents), xylometazoline, and naphazoline hydrochlorides (α-adrenergic receptor agonists), to assess their resistance to radiation sterilization by observing changes in their physicochemical properties. However, there is a research gap, as none of the imidazoline/dimethyl succinate hybrids were investigated by employing any thermal analysis technique to date. Therefore, it is reasonable to carry out the thermal studies with the goal of the detailed thermal characterization of our patented hybrid molecules (**1**–**5**) [1], in which the 2-imidazoline (i.e., 4,5-dihydro-1*H*-imidazole) scaffold is connected through the hydrazinylidene moiety to dimethyl succinate. The mechanism and path of thermal degradation, the thermal behavior, and thermal properties of this class of hybrid materials are unknown.

The novelty of the present paper is to study the thermal decomposition path under inert and oxidizing conditions of imidazoline/dimethyl succinate hybrids **1**–**5**. Hence, a whole set of these molecules is analyzed, using the simultaneous thermogravimetric analysis (TG/DTG) coupled online with Fourier transform infrared (FTIR) spectroscopy and quadrupole mass spectrometry (QMS) analyzers in helium and synthetic air atmospheres, with the purpose of explaining for the first time their thermal decomposition mechanism and path, as well as determining their thermal stability and identifying the volatile degradants under inert and oxidizing conditions. The coupled and simultaneous thermal analysis techniques used in our studies are recommended in the thermal testing of molecular pharmaceuticals, as well as therapeutic agent candidates. They enable the reliable interpretation of any phenomenon occurring with mass and energy changes in the heated sample and the control of its purity [11,12,13,14,15,16,17]. The presented study is a significant contribution to the current state of knowledge on the hitherto unknown thermal decomposition path of 2-imidazoline/dimethyl succinate hybrid molecules. The practical usefulness of our research lies in the fact that the thermal behavior results of these pharmacologically active compounds will be helpful not only in determining the optimal storage and processing conditions for this class of molecules, but also in assessing their impact on the environment during thermal utilization.

In addition, the pharmacological activity of the tested imidazoline/dimethyl succinate hybrids and their toxicity to normal cells are unknown. Therefore, the present biological investigation is aimed at determining the antiproliferative activity of the studied compounds against human tumor epithelial cells, as well as assessing their toxicity to normal cells (being of the same origin) and erythrocytes, which is a novelty in our studies. This should enable the selection of those molecules that are the most active and selective and therefore suitable for further, more extended in vivo studies. The rationale for studying the anticancer activity in this class of molecules is the fact that the heterocyclic hydrazones designed and described earlier showed significant antiproliferative activity in human tumor cells [18]. In turn, the justification for testing the ability to inhibit oxidative hemolysis by these compounds is that the previously described hybrids containing the 4,5-dihydro-1*H*-imidazole template showed the highest antioxidant activity among hydrazones with various heterocyclic rings [19].

## 2. Materials and Methods

### 2.1. Chemicals and Instruments

Fetal bovine serum, RPMI 1640 medium, penicillin-streptomycin (100 U mL^−1^/0.1 mg mL^−1^) stabilized solution, 2,2′-azobis(2-amidinopropane)dihydrochloride (AAPH), hydrogen peroxide, ascorbic acid, 6-hydroxy-2,5,7,8-tetramethylchroman-2-carboxylic acid (Trolox), Triton X-100, and dimethyl sulfoxide were purchased from Sigma-Aldrich (Saint Louis, MO, USA). The 5-Bromo-2′-deoxy-uridine labeling and detection kit III was supplied from Roche Diagnostics GmbH (Mannheim, Germany), while phosphate-buffered saline (PBS; pH 7.4) was from Biomed (Lublin, Poland). The 96-well microtiter plates were bought from Costar (Corning Inc., Glendale, AZ, USA).

A DSC 204 apparatus (Netzsch, Selb, Germany) was employed to evaluate the melting temperatures of the tested compounds. A STA 449 Jupiter F1 instrument (Netzsch, Germany) coupled on-line with a FTIR TGA 585 analyzer (Bruker, Mannheim, Germany) and a QMS 403 C Aëolos (Netzsch, Germany) analyzer was applied to perform simultaneous TG/DTG/FTIR/QMS analyses of the investigated compounds in order to evaluate their thermal stability and to investigate their thermal decomposition path. A FTIR TGA 585 analyzer (Bruker, Germany) and the QMS 403 C Aëolos analyzer (Netzsch, Germany) were used to collect the FTIR and mass spectra of the emitted volatiles. An ELISA reader (BIO-TEK Instruments Inc., Winooski, VT, USA) was employed to measure the optical densities of the samples to assess the anticancer activity and cytotoxicity to normal cells of the tested compounds. A Hitachi U2800 UV-Vis spectrophotometer (Hitachi, Tokyo, Japan) was used to measure the absorbance of the samples to evaluate the hemolytic and antihemolytic properties of the investigated compounds.

### 2.2. The Investigated Compounds (***1***–***5***)

The panel of molecules (**1**–**5**) that was subjected to the detailed thermal studies is shown in Figure 1. Each compound was synthesized in our laboratory by addition of 1-(R-phenyl)-2-hydrazinoimidazoline (where R denotes hydrogen, 4-methyl, 4-methoxy, 4-ethoxy, or 4-chloro group, respectively) to unsaturated dimethyl but-2-ynedioate, according to the general synthesis pathway patented previously [1]. The homogeneity of all the compound samples was checked by TLC. Their structures were established on the basis of consistent spectroscopic data (^1^H NMR and IR spectra) and elemental analyses (within ±0.4 of the theoretical values) [1,20]. The structure of dimethyl 2-{2-[1-(4-methylphenyl)-4,5-dihydro-1*H*-imidazol-2-yl]hydrazinylidene}succinate (**2**) is supported by its ^13^C NMR spectrum [1,20]. All these hybrid molecules found application as the starting materials in the synthesis of pharmacologically active functionalized fused 1,2,4-triazinone derivatives [2,3].

### 2.3. Differential Scanning Calorimetry (DSC)

The melting temperatures of the tested compounds by a use of a DSC analysis were evaluated. The sample with a mass of about 10 mg in an aluminum crucible with a pierced lid from room temperature to 200 °C was heated. The heating rate was 10 K min^−1^. Argon with a flow rate of 40 mL min^−1^ as a furnace atmosphere was applied. The maximum melting temperatures (*T*_peak_) were marked from the DSC curves. 

### 2.4. Simultaneous Thermogravimetric Analysis Coupled On-Line with FTIR and QMS Analyzers (TG/DTG/FTIR/QMS)

The thermal stability and the decomposition path of the tested compounds with the use of a simultaneous TG/DTG/FTIR/QMS analysis were evaluated. The compounds were heated from 40 °C to 750 °C in an inert atmosphere (helium with a flow rate of 40 mL min^−1^) and in an oxidizing atmosphere (a synthetic air with a flow rate of 100 mL min^−1^). The sample with a mass of about 10 mg in an open Al_2_O_3_ crucible was heated. The heating rate was 10 K min^−1^. Simultaneously, the FTIR and mass spectra of the emitted volatiles were collected. The FTIR spectrometer equipped with the IR cell maintained at 200 °C was connected on-line to a STA instrument by a Teflon transfer line with a diameter of 2 mm. It was heated to 200 °C to avoid a condensation process of the volatiles. The FTIR spectra from 600 cm^−1^ to 4000 cm^−1^ with 16 scans per spectrum and with 4 cm^−1^ resolution were collected. The QMS analyzer was connected on-line to a STA instrument by a quartz capillary heated to 300 °C. The QMS instrument was operated under electron ionization of 70 eV. The QMS spectra in the range of 10–150 m/z were gathered.

### 2.5. An Assessment of the Anticancer Activity and Toxicity for Normal Cells of the Tested Compounds

All the imidazoline/dimethyl succinate hybrids, i.e., the unsubstituted compound **1** (the parent structure) and its four derivatives **2**–**5** (differing in a substituent at the phenyl moiety in the *para* position), were assessed for their in vitro growth inhibitory potency against two tumor cell lines: TOV112D (ATCC CRL-11731; human ovarian primary malignant adenocarcinoma cells) and HeLa (ECACC 93021013; human Negroid cervix epitheloid carcinoma cells), as well as cytotoxicity for normal cell line: Vero (ECACC 88020401; African Green Monkey kidney cells). The three reference cell lines that were recruited had the same epithelial origin. Each cell line was grown at 37 °C in the 5% CO_2_ atmosphere in the growth medium containing RPMI 1640 medium with 10% fetal bovine serum and a commercially available penicillin-streptomycin stabilized solution. Each cell culture was plated onto 96-well plates. All the compounds were tested at a concentration of 0.05 mg mL^−1^ after 24-, 48-, and 72 h incubation periods in the bioassay assessing the DNA synthesis and cell proliferation using the commercial 5-bromo-2′-deoxy-uridine labeling and detection kit III, according to the procedure described earlier [21]. The cytotoxicity of each compound was expressed as the percentage growth inhibition, which was calculated from at least three independent experiments.

### 2.6. Study of the Effect of the Investigated Compounds on Red Blood Cells

The evaluation of the hemolytic and antihemolytic activities of the compounds was performed ex vivo on a 4% suspension of red blood cells in phosphate-buffered saline (PBS). Erythrocytes were obtained by the centrifugation (1500 rpm; 10 min; 4 °C) of whole blood from rats (male Wistar rats; 8–9 weeks old; 200–250 g; the Experimental Medicine Centre, Medical University of Lublin, Poland). The hemolytic potential of the compounds (at a concentration of 0.15 mM) was assessed in the hemolytic assay, and their antihemolytic activity was tested in the oxidative hemolysis inhibition assay according to the procedures described earlier [22].

## 3. Results and Discussion

### 3.1. Melting Temperatures of the Tested Compounds

**Figure 2:**
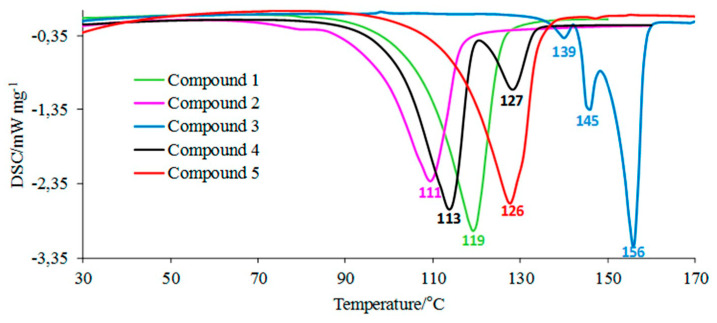
The DSC curves for the tested molecules.

**Figure 3:**
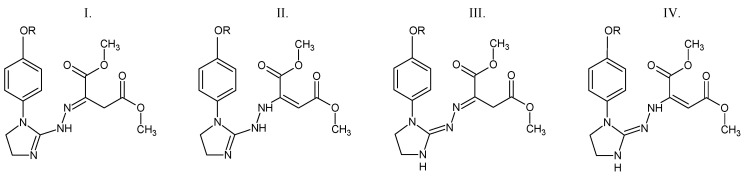
The possible structural isomers of compounds **3** and **4**. I. Dimethyl (2*E*)-2-{2-[1-(4-methoxy/4-ethoxyphenyl)-4,5-dihydro-1*H*-imidazol-2-yl]hydrazinylidene}butanedioate. II. Dimethyl (2*E*)-2-{2-[1-(4-methoxy/4-ethoxyphenyl)-4,5-dihydro-1*H*-imidazol-2-yl]hydrazinyl}but-2-enedioate. III. Dimethyl (2*E*)-2-{(2*Z*)-[1-(4-methoxy/4-ethoxyphenyl)imidazolidin-2-ylidene]hydrazinylidene}butanedioate. IV. Dimethyl (2*E*)-2-{(2*Z*)-2-[1-(4-methoxy/4-ethoxyphenyl)imidazolidin-2-ylidene]hydrazinyl}but-2-enedioate. R = CH_3_ (compound **3**) or CH_2_CH_3_ (compound **4**).

According to the DSC studies, Figure 2, the melting process of compounds **1**, **2**, and **5** is described by one endothermic signal. This means that there is only one assigned isomeric form in their molecules. An interesting melting behavior in the case of two compounds substituted in the *para* position by an electron-donating alkoxy group is observed. The melting process of *para*-methoxy derivative (compound **3**) is described by three endothermic, asymmetrical peaks. The *para*-ethoxy derivative (compound **4**) melts in two temperature ranges. No mass loss at temperatures up to 180 °C is observed. This directly indicates the melting processes of the tested compounds. The previous spectroscopic and chromatographic investigations [1,20] confirmed that compounds **3** and **4** are homogeneous substances of high purity. Hence, it follows that the presence of three or two endothermic DSC peaks is not related to their contamination. Compounds **3** and **4**, due to the attendance of an electron-donating alkoxy group in the *para* position, may have low thermal isomerization barriers. Thus, these DSC peaks most probably referred to the melting of constitutional isomers that can be easily interconvertible during heating (Figure 3). This statement is supported by previous experimental findings. Le Count and Greer [23] were the first who described an analogous behavior with the consecutive and separate melting events during identification of an unknown dimethyl 2-[2-(4,5-dihydro-1*H*-imidazol-2-yl)hydrazinylidene]succinate using a melting point apparatus.

### 3.2. Thermal Stability of the Tested Compounds (Inert Conditions)

**Figure 4:**
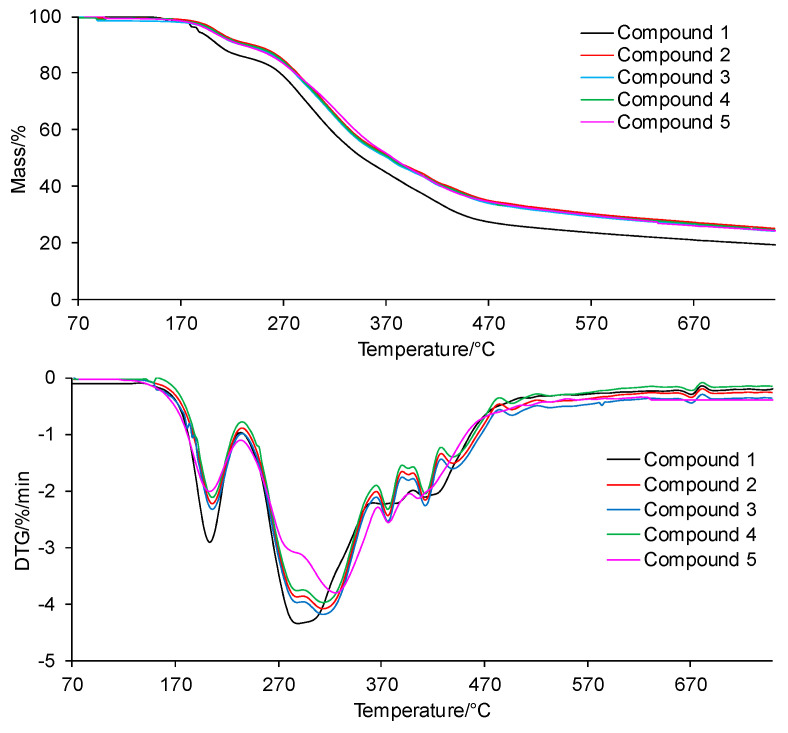
The TG/DTG curves for the tested compounds in inert conditions.

The TG/DTG curves of the tested compounds obtained in inert conditions are presented in Figure 4. In addition, the values of the initial decomposition temperature (*T*_5%_), peak maximum temperature (*T*_max1_), mass loss (Δ*m*_1_), and the residual mass at 750 °C (rm) are placed in Table 1.

According to the TG/DTG curves, the tested compounds are thermally stable up to temperatures of 200–208 °C (*T*_5%_). The thermal resistance of all the tested compounds is similar and does not depend on the type of the substituent at the phenyl moiety in the *para* position or its absence at this moiety. No mass loss due to the decomposition of the tested compounds in this temperature range is observed; only the mass loss related to the evaporation of moisture absorbed by the samples during their storage is observed. 

Above the temperatures of *T*_5%,_ the first decomposition stage is visible. This stage spreads to temperatures of 230–235 °C, with a similar peak maximum temperature (*T*_max1_) for all the tested compounds. A slight mass loss (Δ*m*_1_) within the limits of 9.0–11.0% is observed. Immediately, after the first decomposition stage, the second decomposition stage of the tested compounds begins. This decomposition stage is composed of at least 5–6 unseparated steps. This means that there are many simultaneous processes of breaking bonds in the structure of the tested compounds connected with the emission of volatiles. Moreover, this decomposition stage extends over a wide range of temperatures (from 230–235 °C to 750 °C). The mass loss (Δ*m*_2+_ Δ*m*_2*a*_) is significant and amounts to 65.4–69.6%. This is most likely related to the decomposition of the main skeleton of the tested compounds. In addition, heating the compounds to 750 °C does not cause their complete decomposition. The residual mass (rm) is from 19.4% to 25.1%.

### 3.3. Thermal Stability of the Tested Compounds (Oxidizing Conditions)

**Figure 5:**
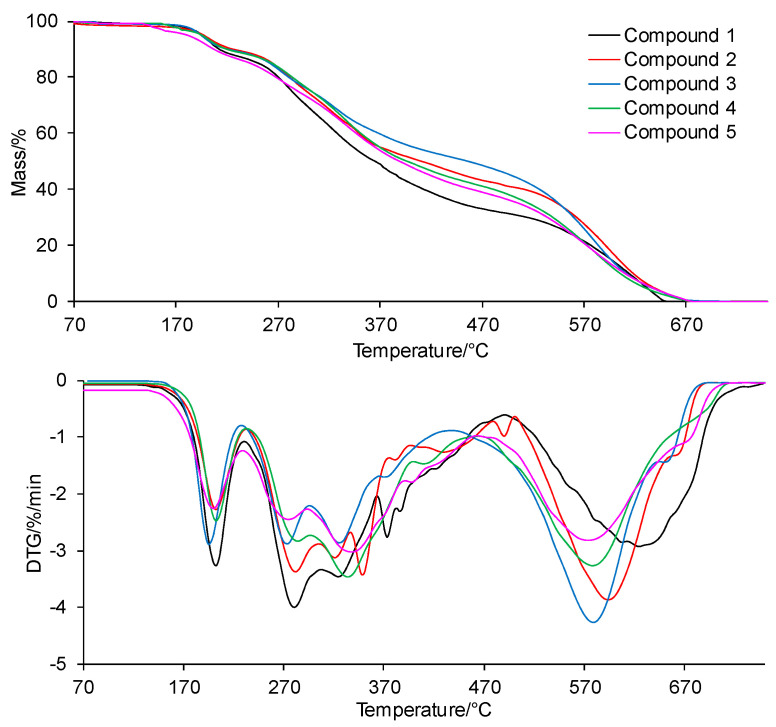
The TG/DTG curves for the tested compounds in oxidizing conditions.

The TG/DTG curves for the compounds collected in oxidizing conditions are presented in Figure 5. In turn, the TG/DTG data are inserted into Table 2.

According to the thermal data, the tested compounds are thermally stable up to the temperatures of 191–197 °C in an oxidizing atmosphere. In light of the current knowledge, pharmaceuticals that are stable at a temperature much higher than ambient temperature can be stored in a wide range of temperatures (in the range of 20–45 °C) without fear of losing their shelf life [24,25]. The mass loss below *T*_5%_ is connected with the evaporation of moisture. In an air atmosphere, the compounds decompose in three main stages. The first decomposition stage starts at temperature above *T*_5%_ and ends at a temperature of 225–235 °C. The mass loss (Δ*m*_1_) is similar to the mass loss observed in an inert atmosphere; it is from 8.9% to 11.7%. The DTG curves show that the first decomposition stage takes place in one main step. This indicates the cleavage of one type of bond present in the structure of the tested compounds. The second decomposition stage spreads from 225–235 °C to 440–500 °C. This stage is composed of at least 5–6 steps. The mass loss (Δ*m*_2+_ Δ*m*_2*a*_) is lower in this stage (from 38.3% to 56.3%) as compared to the mass loss observed in inert conditions. This indicates the possibility of chemical reactions between the core of the intermediates with a higher molecular masses, which are less volatile in this temperature range. The third and last decomposition stage is visible from 440–500 °C to 750 °C with a mass loss (Δ*m*_3_) from 32% to 52.2%. The presence of the third decomposition stage indicates the emission of volatiles formed by the reaction of intermediates or residues with oxygen. The tested compounds decompose completely at 750 °C in an air atmosphere.

### 3.4. Decomposition Course of the Tested Compounds (Inert Conditions)

**Figure 6:**
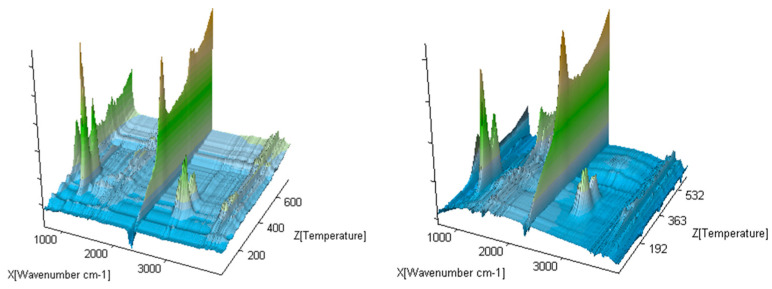
The exemplary gaseous 3D FTIR spectra for compounds **1** and **5**.

The exemplary 3D FTIR spectra for the selected compounds (compound **1** and **4**) are presented in Figure 6.

**Figure 7:**
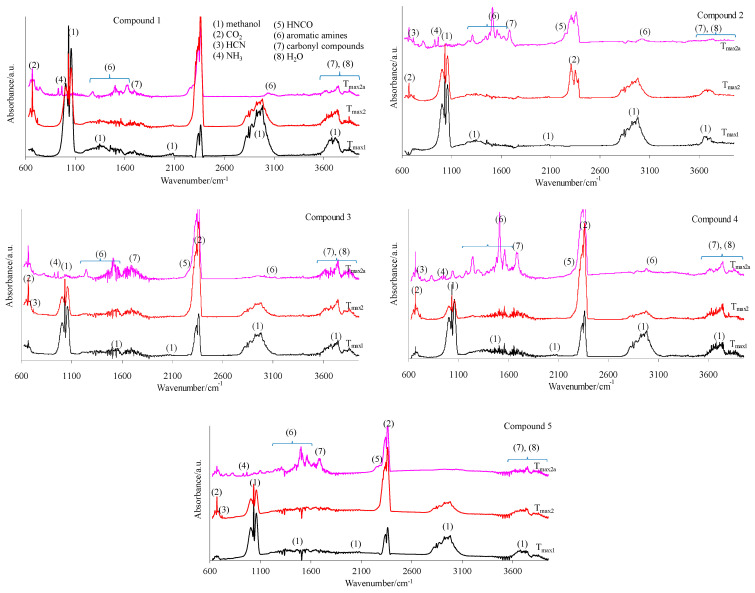
The gaseous FTIR spectra for the tested compounds **1**–**5** (a helium atmosphere).

**Figure 8:**
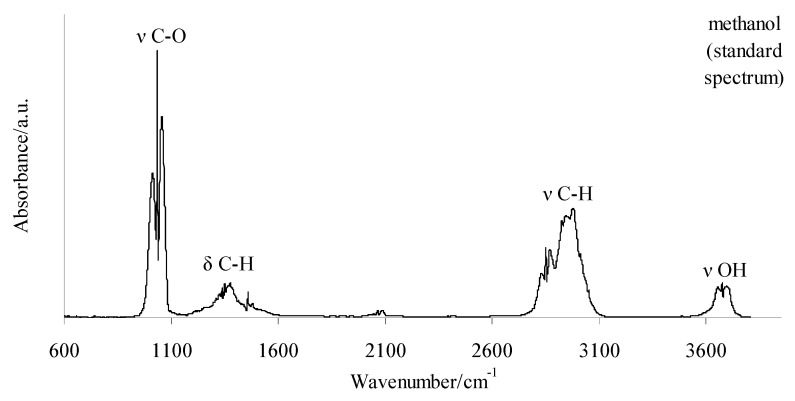
The standard spectrum of gaseous methanol according to the NIST database [26].

**Scheme 1:**
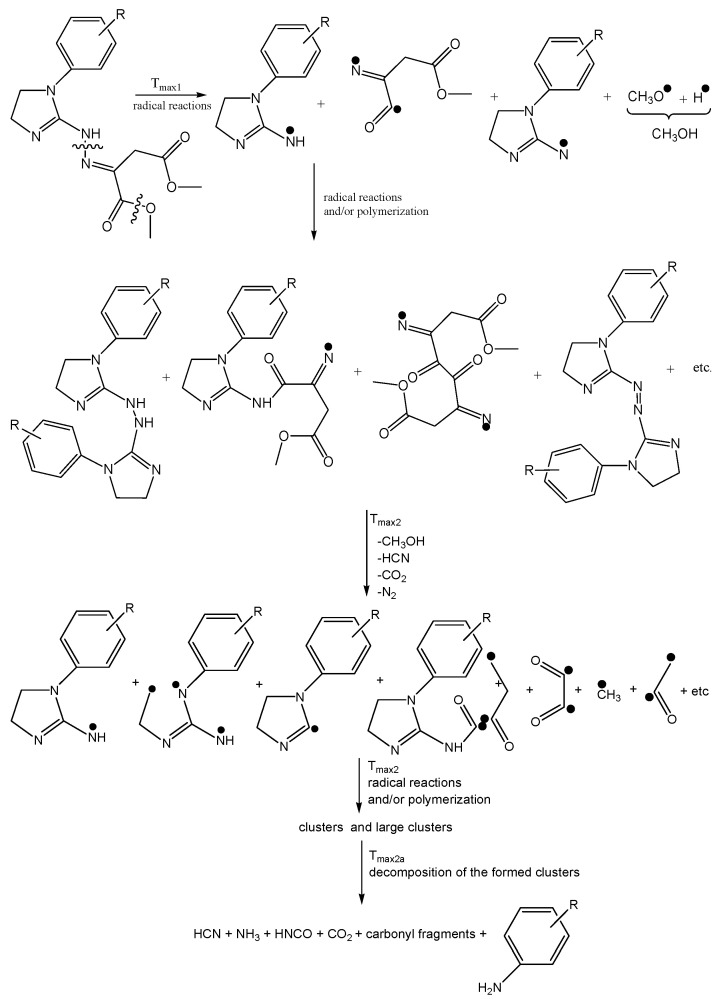
The decomposition path of the tested compounds in inert conditions.

The 2D FTIR spectra for all the tested compounds collected at *T*_max1_, *T*_max2_, and *T*_max2a_ are placed in Figure 7. As it can be seen, the first decomposition stage for all the tested compounds proceeds in the same way. The presence of the stretching vibrations of the C-O at 1002, 1031, and 1060 cm^−1^, the deformation vibrations of the C-H in the range of 1600–1195 cm^−1^, the stretching vibrations of the C-H in the range of 2993–2817 cm^−1^, and the stretching vibrations of the OH above 3550 cm^−1^ at *T*_max1_ is confirmed. This suggests the emission of one major decomposition product. According to the NIST database, this major decomposition product is methanol (Figure 8) [26]. It is formed as a result of the breaking of the ester bonds (C-O) present in the structure of the tested compounds (Scheme 1). As it is confirmed, the mass loss is in the range of 8.7–11.3% in this decomposition stage. Therefore, the cleavage of one of the ester bonds, which requires less energy, is suspected in this decomposition stage.

In turn, at *T*_max2_, the stretching and deformation vibrations characteristic for the groups present in methanol molecules are still observed. In addition to the emission of methanol, the emissions of HCN (the characteristic absorption signal at 713 cm^−1^) and CO_2_ (the absorption signals at 2300–2365 cm^−1^ and 669 cm^−1^) are confirmed based on the FTIR analysis. The mass loss is in the range of 35.3–39.8% in this decomposition stage. This indicates further pyrolysis reactions, such as the cleavage of the second ester bond requiring more energy and the simultaneous cleavage of the N-N and C-C bonds in the structure of the tested compounds.

At *T*_max2a_, the emission of HCN (713 cm^−1^), NH_3_ (the presence of the deformation vibrations of the N-H groups at 931 cm^−1^ and 966 cm^−1^ [27,28,29,30,31,32]), CO_2_ (bands at 2300–2365 cm^−1^ (valence vibration) and 669 cm^−1^ (deformation vibration)), HNCO (bands at 2270–2290 cm^−1^), H_2_O (several, jagged, weak bands in the range of 3450–4000 cm^−1^), aromatic amines, and carbonyl compounds is well visible from the gaseous FTIR spectra. If we look at the initial structure of the tested compounds, the presence of HNCO as the decomposition product is unexpected. Therefore, it is believed that the pyrolysis process proceeds by the radical mechanism. The resulting radicals can react with each other and/or participate in polymerization processes to form intermediate products with higher molecular masses (clusters and large clusters) and with a higher evaporation temperature than the starting compounds, as shown in Scheme 1.

At *T*_max2a_, a slow evaporation of the decomposition products, in particular, aromatic compounds, takes place. The most expected aromatic decomposition product for compound **1** is aniline. Aniline emission by the presence of the stretching vibrations of the C_Ar-H_ at 3054–3100 cm^−1^, the deformation vibrations of the N-H at 1633 cm^−1^, the stretching vibrations of the C_Ar_=C_Ar_ at 1502–1580 cm^−1^, the stretching vibrations of the C-N at 1284 cm^−1^, and the out-of-plane deformation vibrations of the N-H and the C_Ar-H_ at 754–831 cm^−1^ is confirmed. The appearance of the stretching vibrations of the C_Ar-H_ at 3050–3074 cm^−1^, the deformation vibrations of the N-H at 1620–1643 cm^−1^, the stretching vibrations of the C_Ar_=C_Ar_ at 1515–1565 cm^−1^, the stretching vibrations of the C-N at 1297–1317 cm^−1^, and the out-of-plane deformation vibrations of the N-H and the C_Ar-H_ at 784–830 cm^−1^ on the FTIR spectrum for compound **2** confirms the emission of *p*-toluidine. The formation of *p*-anisidine as a result of the decomposition process of compound **3** is visible as the following FTIR absorption bands: the stretching vibrations of the C_Ar-H_ at 3047–3061 cm^−1^, the deformation vibrations of the N-H at 1677 cm^−1^, the stretching vibrations of the C_Ar_=C_Ar_ at 1519–1573 cm^−1^, the stretching vibrations of the C-N at 1253 cm^−1^, and the out-of-plane deformation vibrations of the N-H and the C_Ar-H_ at 757–856 cm^−1^. The absorption signals at 3039–3068 cm^−1^ (the stretching vibrations of the C_Ar-H_), at 1630–1670 cm^−1^ (the deformation vibrations of the N-H), at 1500–1565 cm^−1^ (the stretching vibrations of the C_Ar_=C_Ar_), at 1214–1247 cm^−1^ (the stretching vibrations of the C-N), and at 767–944 cm^−1^ (the out-of-plane deformation vibrations of the N-H and the C_Ar-H_) indicate the formation of *p*-phenetidine for compound **4**. Finally, the emission of *p*-chloroaniline is well visible as a result of the decomposition of compound **5** at the following absorption bands: at 3060 cm^−1^, 1658–1664 cm^−1^, 1504–1565 cm^−1^, 1234–1280, and at 788–848 cm^−1^ [33,34,35]. The formed residues are associated with some of the aromatic compounds with higher masses that do not evaporate at temperatures above 740 °C. A slow mass loss observed from temperatures around 540 °C also confirms the formation of condensed aromatic compounds that decompose gradually to aromatic amines and their derivatives.

**Figure 9:**
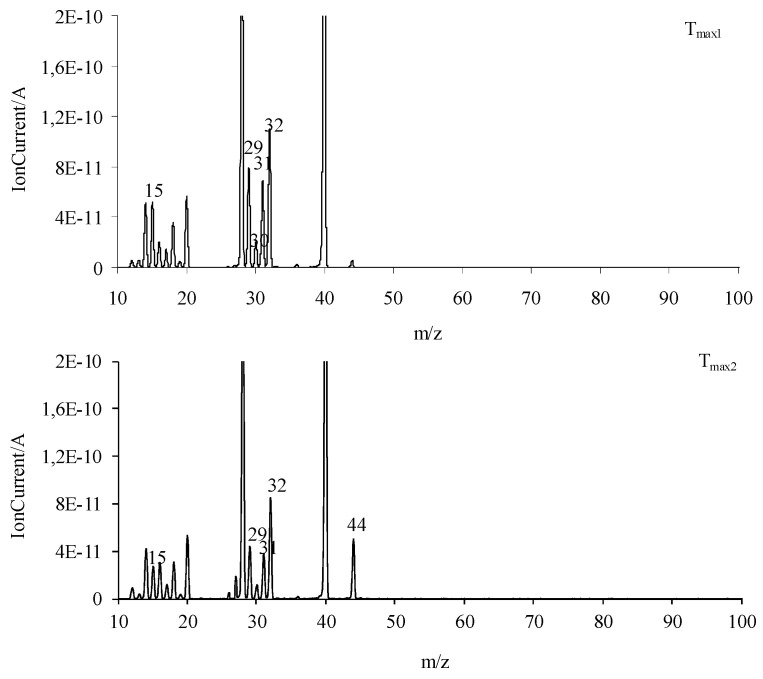
The QMS spectra collected at *T*_max1_ and *T*_max2_ for all the tested compounds.

The obtained FTIR results are also confirmed based on the QMS analyses. As is well visible in Figure 9, at *T*_max1_ and *T*_max2_, the main decomposition product of the tested compounds is methanol. This is confirmed by the presence of the following *m*/*z* ions: 32 (CH_3_OH^+^), 31 (CH_3_O^+^), 30 (CH_2_O^+^), 29 (CHO^+^), and 15 (CH_3_^+^). In addition to the emission of methanol, at *T*_max1_, the formation of a small amount of CO_2_ (*m*/*z* 44 CO_2_^+^, 28 CO^+^) is confirmed. The emission of methanol and the beginning of HCN emission at *T*_max2_ is observed. HCN emission by the attendance of the *m*/*z* ions 27 (HCN^+^) and 26 (CN^+^) is confirmed. In addition, the emission of N_2_ at *T*_max2_ is very likely. The N_2_ molecule is a symmetrical molecule invisible to the FTIR radiation. However, the structure of the formed intermediates in polymerization processes or other chemical reactions during the heating of the tested compounds and the presence of *m*/*z* ions 28 (N_2_^+^) and 14 (N^+^) in the QMS spectrum suggest the emission of nitrogen.

At *T*_max2a_, the formation of HCN, CO_2_, and H_2_O is also observed. The new decomposition products visible only at *T*_max2a_ are ammonia (*m*/*z* 17 (NH_3_^+^), 16 (NH_2_^+^) and 15 (NH^+^)), carbonyl compounds (*m*/*z* ions: 43 (CH_3_CO^+^), 42 (CH_2_CO^+^), 41 (CHCO^+^), 40 (CCO^+^), 30 (H_2_CO^+^), 29 (HCO^+^) and 28 (CO^+^)), HNCO (*m*/*z* ions: 43 (HNCO^+^), 42 (NCO^+^)), and aromatic compounds. Additionally, further emission of nitrogen at *T*_max2a_ cannot be ruled out due to the presence of *m*/*z* ions 28 and 14.

**Figure 10:**
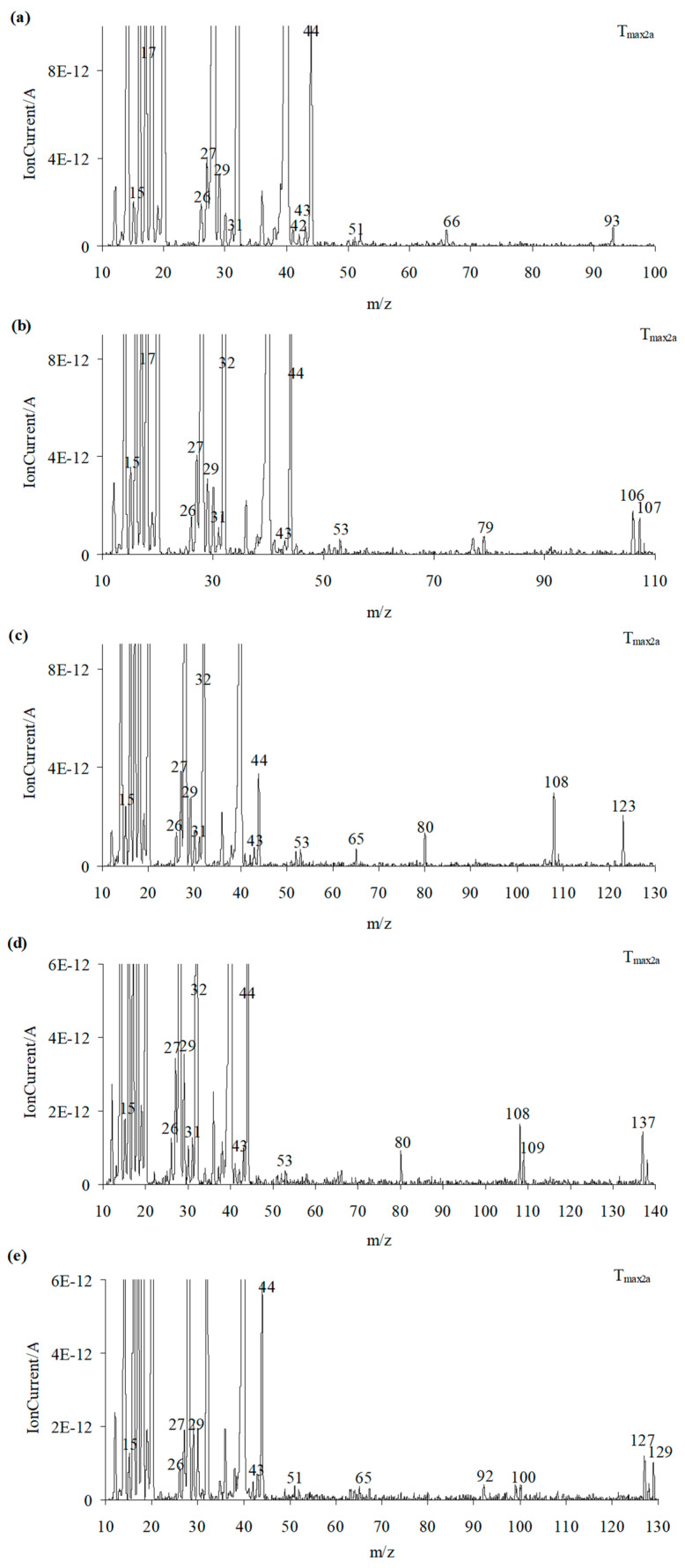
The QMS spectra at *T*_max2a_ for compound **1** (**a**), compound **2** (**b**), compound **3** (**c**), compound **4** (**d**), and compound **5** (**e**).

As is well visible from Figure 10a, the emission of aniline by the presence of the *m*/*z* ions 93 (C_6_H_7_N^+^), 92 (C_6_H_6_N^+^), 66 (C_5_H_6_^+^), 65 (C_5_H_5_^+^), 51 (C_4_H_3_^+^), and 39 (C_3_H_3_^+^) in the QMS spectra is confirmed. By the ionization of aniline, the elimination of one HCN molecule from its structure can occur. This leads to additional emission of HCN and the formation of the *m*/*z* ion 65 (cyclopentadienyl ion), the presence of which is confirmed from the QMS spectra collected at *T*_max2a_. The QMS data confirm the creation of *p*-toluidine as a result of the decomposition of compound **2**. Its presence is visible as the following *m*/*z* ions: 107 (C_7_H_9_N^+^), 106 (C_7_H_8_N^+^), 79 (C_6_H_7_^+^), 78 (C_6_H_6_^+^), 77 (C_6_H_5_^+^), and 53 (C_4_H_5_^+^). Additionally, in this case, the ionization of *p*-toluidine leads to the formation of the aminotropylium ion (*m*/*z* 106). This ion loses one HCN molecule and forms the C_6_H_6_^+^ (*m*/*z* 78) [36], Figure 10b. The decomposition of compound **3** results in the emission of *p*-anisidine as a main aromatic volatile product at *T*_max2a_. The ionization of *p*-anisidine causes the formation of *m*/*z* ions: 123 (C_7_H_9_ON^+^) at *T*_max2a_. The homolytic cleavage of the ether bond (-O-CH_3_) results in a loss of the methyl group and the formation of *m*/*z* ion 108 (C_6_H_6_ON^+^). In addition, the other m/z ions characteristic of *p*-anisidine, such as 80 (C_5_H_6_N^+^), 65 (C_5_H_5_^+^), 53 (C_4_H_5_^+^), 52 (C_4_H_4_^+^), and 39 (C_3_H_3_^+^), confirm its formation (Figure 10c). Compound **4** breaks down to form the aromatic compound *p*-phenetidine: *m*/*z* 137 (C_8_H_11_ON^+^), 109 (C_8_H_6_O^15^N^+^), *m*/*z* 108 (C_8_H_6_ON^+^), 81 (C_5_H_6_^15^N^+^), and 80 (C_5_H_6_N^+^) (Figure 10d). Finally, in the case of compound **5**, the emission of *p*-chloroaniline is confirmed by the presence of the *m*/*z* ions: 129 (C_6_H_6_N^37^Cl^+^), 127 (C_6_H_6_N^35^Cl^+^), 92 (C_6_H_5_NH^+^), 65 (C_5_H_5_^+^), 64 (C_5_H_4_^+^), and 63 (C_5_H_3_^+^) (Figure 10e).

### 3.5. Decomposition Course of the Tested Compounds (Oxidizing Conditions)

**Figure 11:**
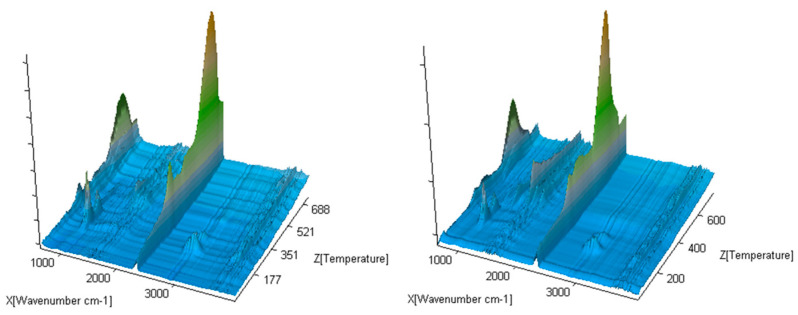
The exemplary gaseous 3D FTIR spectra for compounds **1** and **4**.

**Figure 12:**
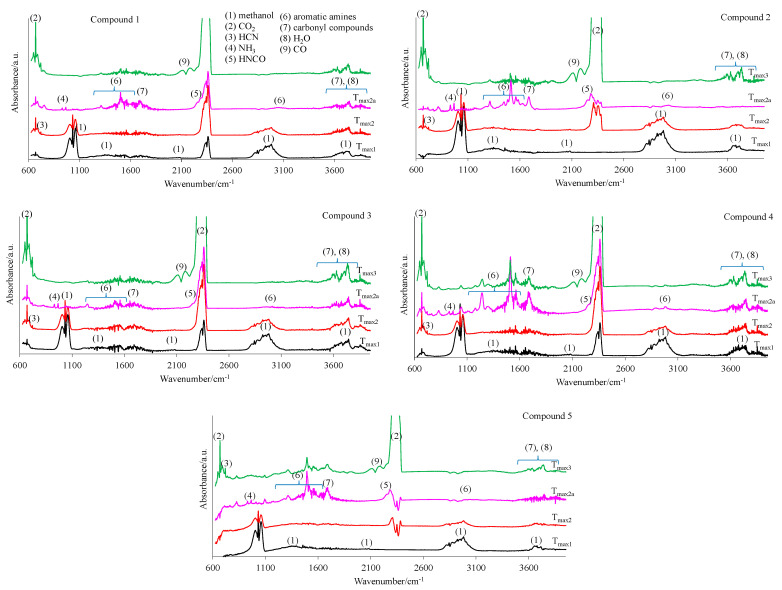
The gaseous FTIR spectra for the tested compounds **1**–**5** (a synthetic air atmosphere).

**Figure 13:**
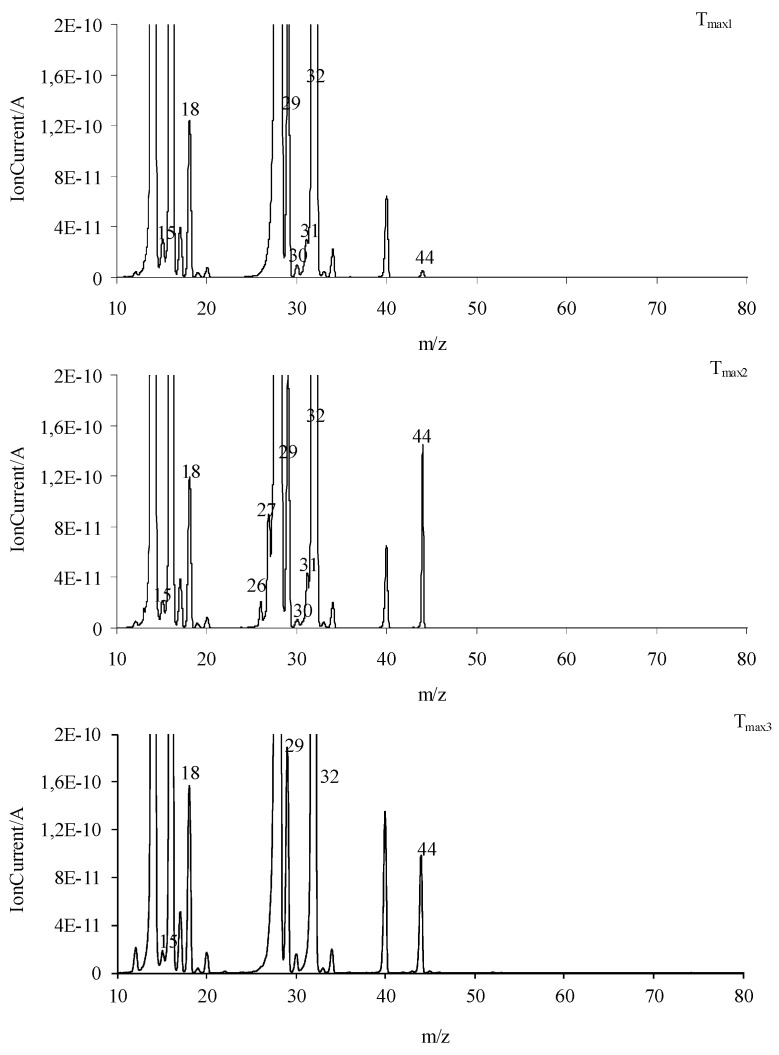
The QMS spectra at *T*_max1_, *T*_max2_, and *T*_max3_ for all the tested compounds (common graphs).

**Figure 14:**
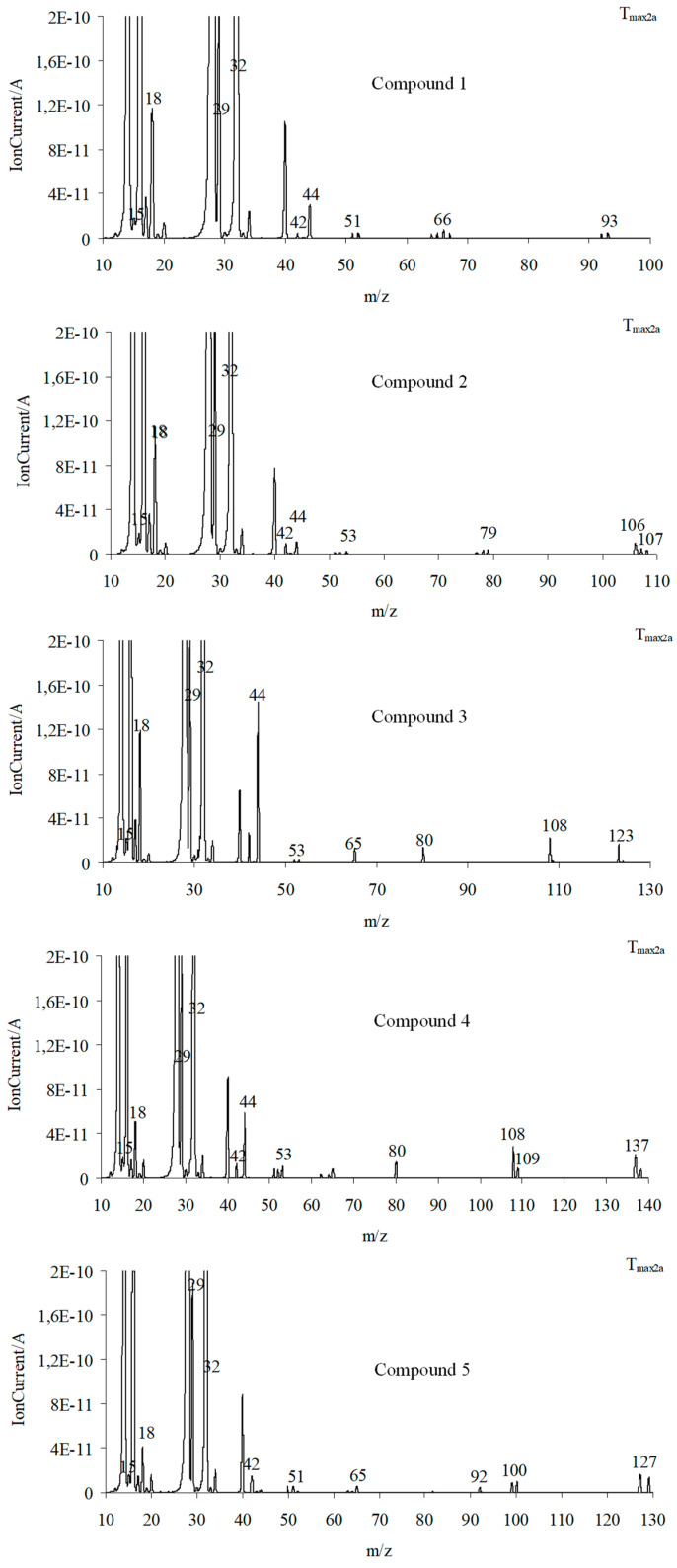
The QMS spectra for the volatiles emitted at *T*_max2a_ in a synthetic air atmosphere.

The exemplary gaseous 3D FTIR spectra are given in Figure 11. The extracted 2D gaseous FTIR spectra at *T*_max1_, *T*_max2_, *T*_max2a_, and *T*_max3_ are presented in Figure 12. In addition, the QMS spectra collected at *T*_max1_, *T*_max2_, *T*_max2a_, and *T*_max3_ are placed in Figure 13 and Figure 14.

The main decomposition product of the tested compounds is methanol (FTIR: the stretching vibrations of the C-O at 1002–1060 cm^−1^, the deformation vibrations of the C-H at 1600–1195 cm^−1^, the stretching vibrations of the C-H at 2993–2817 cm^−1^, and the stretching vibrations of the OH above 3550 cm^−1^; *m*/*z*: 32 (CH_3_OH^+^), 31 (CH_3_O^+^), 30 (CH_2_O^+^), 29 (CHO^+^), and 15 (CH_3_^+^)) in an oxidizing atmosphere. At *T*_max1_, the emission of small amounts of CO_2_ and H_2_O is also observed. These results indicate the same decomposition products in oxidizing and inert atmospheres. 

In the second decomposition stage (*T*_max2_), the emission of methanol, HCN (FTIR: the absorption signal at 713 cm^−1^; *m*/*z*: 27 (HCN^+^) and 26 (CN^+^)), CO_2_ (FTIR: the peaks at 2300–2365 cm^−1^ and 669 cm^−1^; *m*/*z*: 44 CO_2_^+^, 28 CO^+^)) and H_2_O (FTIR: the signals at 3450–4000 cm^−1^; *m*/*z*: 18 H_2_O^+^), 17 (OH^+^), and 16 (O^+^) is confirmed. The QMS spectra show the presence of the characteristic m/z ions for N_2_ emission, namely 28 and 14. This leads to the conclusion that the first and the second decomposition stages of the tested compounds in the presence of oxygen proceed in the same or similar way as compared to these observed without access to oxygen. In these two stages, the C-O, C-C, and N-N bonds are broken. As a result, the emission of the previously mentioned low-mass products and the clusters and large cluster formations in radical reactions are observed. The creation of ammonia (FTIR: two bands at 931cm^−1^ and 966 cm^−1^; m/z: 17 (NH_3_^+^), 16 (NH_2_^+^) and 15 (NH^+^)), carbonyl fragments (the absorption band at 1706–1787 cm^−1^; *m*/*z* ions: 43 (CH_3_CO^+^), 42 (CH_2_CO^+^), 41 (CHCO^+^), 40 (CCO^+^), 29 (HCO^+^), and 28 (CO^+^)), HNCO (FTIR: peaks at 2270–2290 cm^−1^, *m*/*z* ions: 43 (HNCO^+^), 42 (NCO^+^)), HCN (FTIR: peak at 713 cm^−1^, *m*/*z* ions: 27 (HCN^+^) and 26 (CN^+^)) and aromatic compounds is confirmed from the temperature *T*_max2a_. 

Finally, the third decomposition stage (*T*_max3_) above a temperature of 460 °C is observed in an oxidizing atmosphere. In this stage, mainly the emission of CO_2_ (FTIR: the peaks at 2300–2365 cm^−1^ and 669 cm^−1^; m/z: 44 CO_2_^+^, 28 CO^+^), CO (FTIR: 28 CO^+^), and H_2_O (FTIR: the signals at 3450–4000 cm^−1^; m/z: 18 H_2_O^+^), 17 (OH^+^), and 16 (O^+^)) [26,36] is indicated (Figure 13 and Figure 14). The emission of these inorganic gases is the result of the oxidation and combustion processes of aromatic compounds and higher-molecular mass intermediate compounds formed as a result of the radical reactions. Based on the obtained results, it can be assumed that the decomposition mechanism of the tested compounds in the presence of oxygen is similar to their decomposition in an oxygen-free atmosphere. This means that the attendance of oxygen does not affect the chemical reactions between the formed intermediate products, including radicals. The same gaseous decomposition products are released in both atmospheres. This confirms the same decomposition path of the tested compounds in the presence or in the absence of oxygen. 

### 3.6. Antitumor Activity and Cytotoxicity to Normal Cells of the Investigated Compounds

**Figure 15:**
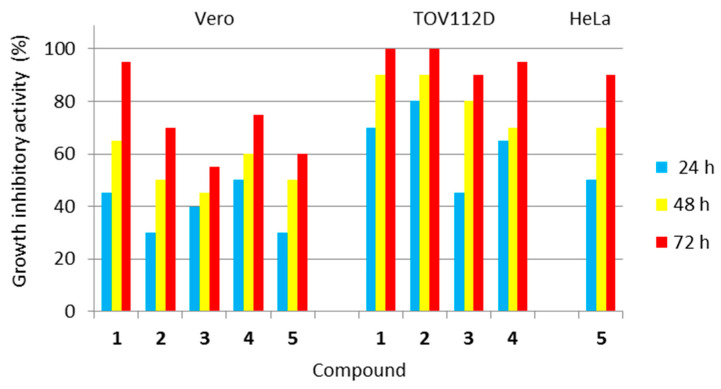
Antiproliferative activity of all the investigated compounds (**1**–**5**) against human tumor cells, as well as their cytotoxicity towards normal cells. Normal cell line: Vero—(ECACC 88020401)—African green monkey kidney cells. Tumor cell lines: TOV112D (ATCC CRL-11731)—human ovarian primary malignant adenocarcinoma cells; and HeLa (ECACC 93021013)—human Negroid cervix epitheloid carcinoma cells. Compound **5** was not active against TOV112D cells, while compounds **1**–**4** were not active against HeLa cells.

The investigated class of hybrid materials includes the unsubstituted compound (the parent structure **1**) and its *para*-substituted derivatives (**2**–**5**). The antiproliferative activity of all the compounds (**1**–**5**) was assessed against two tumor cell lines, i.e., human ovarian primary malignant adenocarcinoma (TOV112D) cells and human Negroid cervix epitheloid carcinoma (HeLa) cells. In turn, their cytotoxicity to normal cells was evaluated using the African green monkey kidney (Vero) cell line. The vast majority of imidazoline-based hybrids with dimethyl succinate (**1**–**4**) revealed the remarkable in vitro growth inhibitory potency against human tumor cells of the ovary, suggesting that unsubstituted electron reach phenyl moiety or electron-donating alkyl (such as methyl) and alkoxy (such as methoxy or ethoxy) groups at the *para* position of this moiety are necessary for the potency against this type of tumor cell. In turn, only one imidazoline-based hybrid with dimethyl succinate (**5**) proved to be effective against human tumor cells of the cervix, suggesting that an electron-withdrawing chloro group placed *para* to the benzene ring is necessary for the potency against this type of tumor cell. The antitumor activity of all the studied compounds (**1**–**5**) is presented in Figure 15.

Analyzing the activity of the *para*-substituted derivatives in relation to the parent compound, it was shown that introducing an electron-donating methyl group *para* to the phenyl moiety in **2** was successful in terms of selectivity due to a decrease in cytotoxicity towards normal Vero cells after all incubation periods. This modification was also responsible for a slight increase, only after 24 h of incubation, in antiproliferative activity against ovarian cancer cells when compared to **1**. Placing an electron-donating methoxy group *para* to the phenyl moiety in **3** was beneficial due to a decrease in cytotoxicity towards normal Vero cells, especially after 48 and 72 h incubation periods. Simultaneously, this substitution was responsible for a slight decrease in antiproliferative activity against ovarian cancer cells after 48 and 72 h of incubation. Introducing an electron-donating ethoxy group *para* to the phenyl moiety in **4** was favourable due to a significant decrease in cytotoxicity towards normal Vero cells after 72 h of incubation. Unfortunately, this modification was responsible for a decrease in antiproliferative activity against ovarian cancer cells, especially after 48 h of incubation. In turn, placing an electron-withdrawing chloro group *para* to the phenyl moiety in **5** was advantageous for normal Vero cells (decreasing the cytotoxicity) and cervical cancer cells (evoking the antitumor potency), but resulted in a loss of antiproliferative activity against ovarian cancer cells, when compared to the parent compound (**1**).

The methoxy and chloro groups in *para*-substituted derivatives were found to be the best choice of substitution patterns due to their increased selectivity. 

Concluding, the two imidazoline-based hybrids with dimethyl succinate, i.e., those containing the *para*-methoxy and *para*-chloro substituent at the phenyl moiety (**3** and **5**, respectively), proved to be the least toxic to normal Vero cells. Therefore, these compounds may be utilized in further, more extended in vivo investigations. 

### 3.7. Assessment of the Effect of the Tested Compounds (***1***–***5***) on Red Blood Cells

Taking into account the high thermal stability and biological activity of the title compounds, their impact on red blood cells, the most numerous cells in the living organism, was also assessed.

Evaluation of the hemolytic potential of the synthesized compounds is an important part of the assessment of their toxicity. Mammal non-nucleated red blood cells represent a good model for studying this cytotoxicity. Therefore, we performed ex vivo hemolysis test to determine possible interactions of the tested imidazoline/dimethyl succinate hybrids with blood components as a necessary part of their biocompatibility in the preclinical phase of drug development. When examining their impact on erythrocytes, it turned out that none of the tested compounds promoted any significant hemolytic effects. We found that the hemolytic activity of the molecules was less than 5% compared to Triton X-100 (a positive control with 100% hemolytic activity); therefore, all the tested hybrid materials are safe for red blood cells. This hemocompatibility of the compounds is an important feature of drug candidates.

Additionally, we assessed the ability of the title compounds to inhibit oxidative hemolysis. For this purpose, red blood cells were pre-treated with the test compounds and then exposed to reactive oxygen species, such as AAPH-derived peroxyl radicals or hydrogen peroxides. It was shown that, among all the imidazoline/dimethyl succinate hybrids tested, compounds **3** (the *p*-methoxy derivative) and **1** (the parent structure) showed the highest ability to protect the erythrocyte cells from AAPH/H_2_O_2_-induced hemolysis. The antihemolytic activities of these compounds (**3** and **1**) were comparable to the activities of standard antioxidants, showing 108% and 99% of ascorbic acid activity (in the case of AAPH-induced hemolysis), and 102% and 99% of Trolox activity (in the case of H_2_O_2_-induced hemolysis). This proves that these hybrid materials are able to effectively protect red blood cells from the oxidative stress-induced damage.

### 3.8. Predicting Molecular Targets for the Investigated Compounds (***1***–***5***)

On the basis of the Therapeutic Target Database (TTD) (http://db.idrblab.net/ttd/, accessed on 6 May 2023), it was found that none of imidazoline/dimethyl succinate hybrids (**1**–**5**) had a Tanimoto coefficient above 0.6. This means that each molecule is not structurally similar to any approved/investigational pharmaceutic.

In order to assess the possible molecular targets for the investigated compounds (**1**–**5**), the Molinspiration Cheminformatics free web server (available online at www.molinspiration.com, accessed on 6 May 2023) was used. Results of this in silico screening are presented as bioactivity scores (Table 3). The higher the value of bioactivity score, the higher the probability of the molecule to be active. The imidazoline-based hybrids with dimethyl succinate (**1**–**5**) were virtually screened as potential G-protein-coupled receptor (GPCR) ligands, ion channel modulators, enzyme inhibitors, protease inhibitors, kinase inhibitors, and nuclear receptor ligands. It turned out that these molecules can act on a variety of molecular targets. Taking into account the bioactivity scores shown in Table 3, they have the highest probability to be moderately active as ligands modulating GPCRs and ion channels.

## 4. Conclusions

The performed TG/FTIR/QMS studies confirmed a similar thermal stability of the tested imidazoline/dimethyl succinate hybrids (**1**–**5**) in inert (200–208 °C) and in oxidizing (191–197 °C) conditions. They were thermally stable at temperatures much higher than ambient conditions. Such thermostability of these compounds will be of importance in the case of their approval as pharmaceutics. It can be assumed that no special recommendations regarding the conditions of their storage and processing will be required. In addition, their thermal resistance was independent of the type of substituent attached in the *para* position of the phenyl moiety or its absence at this moiety. The tested compounds decomposed in two main stages in inert conditions and in three main stages in oxidizing conditions. The first decomposition stage was connected with a breaking of one ester bond, and thus the emission of the methanol molecule and the formation of radical fragments in both furnace atmospheres. However, the second decomposition stage was associated with multi-step processes. The cleavage of the second ester bond, N-N, C-C bonds, and radical reactions between intermediate fragments resulted in the emission of volatiles such as CH_3_OH, NH_3_, HCN, HNCO, aromatic amines, carbonyl fragments, CO_2_, and H_2_O in both atmospheres and the formation of the residue in inert conditions. In oxidizing conditions, the reactions between residue and oxygen led to the full decomposition of the tested compounds and the emission of CO_2_, CO, and H_2_O.

These studies confirmed the same radical decomposition mechanism of the tested compounds in both furnace atmospheres. They also proved that the presence of oxygen did not change the decomposition course of these compounds but only reduced the initial decomposition temperature and influenced the total degradation of the tested compounds (oxidation and combustion of the residue). 

Taking into account the results of biological studies, the two hybrid materials (**3** and **5**), as the most selective and therefore the best compounds, may be utilized in further, more extended in vivo investigations. In addition, it was proven that the molecules **1** and **3** were able to effectively protect red blood cells from the oxidative stress-induced damage. Therefore, these molecules may be utilized as lead structures for the development of potential adjuvant agents in the prevention of free radical-mediated diseases.

## Figures and Tables

**Table 1:** The TG/DTG data collected in inert conditions.

Sample	Degradation Process						
	First Decomposition Stage			Second Degradation Stage			
	*T*_5%_/°C	*T*_max1_/°C	Δ*m*_1_/%	*T*_max2_/°C	*T*_max2a_/°C	Δ*m*_2+_ Δ*m*_2*a*_/%	rm/%
**1**	200	206	11.0	289/305	378/414	69.6	19.4
**2**	208	209	9.5	287/313	377/413/439/495	65.4	25.1
**3**	206	209	9.0	289/317	379/414/499	66.9	24.1
**4**	203	209	9.7	290/319	377/414/444/501	65.7	24.6
**5**	201	207	9.8	278/326	378/407	65.9	24.3

**Table 2:** The TG/DTG data collected in oxidizing conditions.

Sample	Degradation Process							
	First Decomposition Stage			Second Degradation Stage			Third Degradation Stage	
	*T*_5%_/°C	*T*_max1_/°C	Δ*m*_1_/%	*T*_max2_/°C	*T*_max2a_/°C	Δ*m*_2+_ Δ*m*_2*a*_/%	*T*_max3_/°C	Δ*m*_3_/%
**1**	195	202	11.7	281/325	373/386	56.3	625	32.0
**2**	197	203	8.9	282/321	349/381/490	50.4	594	40.7
**3**	194	196	9.5	273/326	372	38.3	579	52.2
**4**	195	203	8.7	284/334	410	49.1	578	42.2
**5**	191	200	10.6	275/338	398	49.2	574	40.2

**Table 3:** Prediction of the bioactivity score for various molecular targets.

Compound	Bioactivity Score					
	GPCR Ligand	Ion Channel Modulator	Enzyme Inhibitor	Protease Inhibitor	Kinase Inhibitor	Nuclear Receptor Ligand
**1**	−0.17	−0.24	−0.34	−0.46	−0.70	−0.81
**2**	−0.20	−0.31	−0.39	−0.48	−0.72	−0.80
**3**	−0.18	−0.28	−0.36	−0.44	−0.67	−0.74
**4**	−0.23	−0.30	−0.39	−0.46	−0.71	−0.70
**5**	−0.16	−0.24	−0.37	−0.47	−0.69	−0.79

GPCR—G-protein-coupled receptor. The bioactivity score interpretation for organic molecules: above 0.00—active; in the range from −5.00 to 0.00—moderately active; and below −5.00—inactive [37,38,39].

## Data Availability

The data presented in this study are available on request from the authors.
